# RCAN1 Regulates Mitochondrial Function and Increases Susceptibility to Oxidative Stress in Mammalian Cells

**DOI:** 10.1155/2014/520316

**Published:** 2014-06-09

**Authors:** Heshan Peiris, Daphne Dubach, Claire F. Jessup, Petra Unterweger, Ravinarayan Raghupathi, Hakan Muyderman, Mark P. Zanin, Kimberly Mackenzie, Melanie A. Pritchard, Damien J. Keating

**Affiliations:** ^1^Molecular and Cellular Neuroscience Group, Department of Human Physiology and Centre for Neuroscience, Flinders University, Adelaide, SA 5042, Australia; ^2^Department of Biochemistry and Molecular Biology, Monash University, Melbourne, VIC 3800, Australia; ^3^Islet Biology Laboratory, Department of Anatomy and Histology and Centre for Neuroscience, Flinders University, Adelaide, SA 5042, Australia; ^4^Discipline of Medicine, University of Adelaide, Adelaide, SA 5001, Australia; ^5^Glial Cell Biology and CNS Repair Laboratory, Department of Medical Biochemistry and Centre for Neuroscience, Flinders University, Adelaide, SA 5042, Australia

## Abstract

Mitochondria are the primary site of cellular energy generation and reactive oxygen species (ROS) accumulation. Elevated ROS levels are detrimental to normal cell function and have been linked to the pathogenesis of neurodegenerative disorders such as Down's syndrome (DS) and Alzheimer's disease (AD). RCAN1 is abundantly expressed in the brain and overexpressed in brain of DS and AD patients. Data from nonmammalian species indicates that increased RCAN1 expression results in altered mitochondrial function and that RCAN1 may itself regulate neuronal ROS production. In this study, we have utilized mice overexpressing RCAN1 (RCAN1^ox^) and demonstrate an increased susceptibility of neurons from these mice to oxidative stress. Mitochondria from these mice are more numerous and smaller, indicative of mitochondrial dysfunction, and mitochondrial membrane potential is altered under conditions of oxidative stress. We also generated a PC12 cell line overexpressing RCAN1 (PC12^RCAN1^). Similar to RCAN1^ox^ neurons, PC12^RCAN1^ cells have an increased susceptibility to oxidative stress and produce more mitochondrial ROS. This study demonstrates that increasing RCAN1 expression alters mitochondrial function and increases the susceptibility of neurons to oxidative stress in mammalian cells. These findings further contribute to our understanding of RCAN1 and its potential role in the pathogenesis of neurodegenerative disorders such as AD and DS.

## 1. Introduction

The RCAN1 (regulator of calcineurin 1) gene is located in the 21q22.1-q22.2 region of human chromosome 21 and is expressed primarily in brain, heart, and skeletal muscle [1] and in endocrine tissues including the adrenal gland [2] and pancreas [3]. RCAN1 is best characterized as an endogenous inhibitor of the phosphatase calcineurin [4]. It exists as two protein isoforms, named RCAN1.1 and RCAN1.4 depending on exon at which transcription is initiated. Calcineurin itself regulates the transcription of RCAN1.4, but not RCAN1.1, due to the presence of NFAT initiation sites upstream of exon 4 [5]. In this respect, RCAN1.4 forms a negative feedback loop in cells regulating calcineurin-dependent dephosphorylation. Furthermore, RCAN1.1, but not RCAN1.4, expression, is induced by thyroid hormone [5]. These two isoforms may have independent cell functions as our own research has demonstrated that RCAN1.1 regulates exocytosis [6, 7], learning, memory and synaptic transmission [8], *β*-cell function and insulin secretion [3], and immune cell function [9]. Short- or long-term expression of RCAN1.1 protects against or promotes, respectively, neuronal cell apoptosis in response to oxidative stress [10]. RCAN1.4, while typically being expressed at lower levels than RCAN1.1, may also have independent functions. RCAN1.4 gene expression is more highly upregulated in response to oxidative stress, although this does not correlate with increased protein expression [11], and upregulation of RCAN1.4 is observed in peri-infarct cortex of stroke [12]. Overexpression of RCAN1.4 exacerbates Ca^2+^-induced apoptosis in neurons [13], while RCAN1.4 knockdown attenuates cell growth through the inhibition of Ras signaling [14] and protects against apoptosis [15].

RCAN1.1 is overexpressed in the brains of both DS and AD individuals [2, 4, 16]. RCAN1 is postulated to provide a link between chronic stress and neurodegeneration [17] and have an essential role in the pathogenesis of AD by linking amyloid-*β* toxicity and tau hyperphosphorylation [18]. The pathological hallmarks of AD are amyloid plaques and hyperphosphorylated tau with neurofibrillary tangles. More than 25 AD clinical trials targeting amyloid *β* have failed to show any significant reduction in disease burden [19]. The fact that trials have shown clearance of amyloid *β* deposits in the brains of AD subjects without impact on either clinical disease progression or progression of tau aggregation pathology [20] indicates that while amyloid pathology exists in AD brains, it may not be the primary driver of sporadic AD. Growing evidence supports the notion that tau can drive AD and amyloid toxicity [21] and higher RCAN1, as observed in AD brains [16], increases the phosphorylation of tau [17]. This mechanism likely occurs through the increased calcineurin inhibition in AD brains [22] and calcineurin is responsible for tau dephosphorylation [23]. Furthermore, RCAN1 expression correlates strongly with the amount of neurofibrillary tangles observed in AD brains [24].

It is worth noting that most DS individuals experience seizures and all are mentally impaired and develop Alzheimer's-like neuropathology by their mid-30's, characterized by *β*-amyloid peptide-containing neuritic plaques, tau-containing neurofibrillary tangles, basal forebrain cholinergic neuron degeneration, and dementia [25]. Furthermore, neuronal oxidative stress and mitochondrial dysfunction are early hallmarks of both DS [26] and AD [27]. Mitochondria are an essential intracellular organelle responsible for cellular energy generation. Mitochondria are also the major site of reactive oxygen species (ROS) which are produced primarily as a byproduct of oxidative phosphorylation. Elevated ROS levels and impaired mitochondrial function are seen in DS cortical neurons in culture [28, 29] as is ROS accumulation in brain tissue from individuals with DS [30] and from Ts65Dn mice [31]. Mitochondrial size and number are also altered in hippocampal neurons from AD patients [27] and oxidative damage is a prominent and early feature of vulnerable neurons in AD [32, 33]. Mitochondrial respiration is impaired in transgenic AD mice overexpressing both amyloid-*β* and tau, with tau preferentially impairing complex I of the respiratory chain and amyloid-*β* blocking complex IV-dependent respiration [34]. A significant decrease of complex IV activity is observed in the cortical regions of AD brains [35]. Additionally, RCAN1 expression is induced in neurons by both amyloid-*β* and oxidative stress [2, 36]. RCAN1 also alters cellular susceptibility to oxidative stress as neurons with no RCAN1 expression display an increased resistance to damage by ROS [11].

Evidence from* Drosophila melanogaster* suggests that* nebula*, the homolog of mammalian RCAN1, is involved in the regulation of mitochondrial function [37]. Altered expression of* nebula* in* Drosophila *neurons results in reduced mitochondrial size, increased mitochondrial number, and increased reactive oxygen species generation [37]. However, whether the chronic overexpression of RCAN1, as occurred in DS and AD brains, regulates mitochondria function in mammalian cells remains unknown. In this study, we have utilised multiple mammalian cell types commonly used in the study of neuronal function to assess the effect of chronically increased RCAN1 expression on mitochondrial morphology and function, including ROS production, and the susceptibility of neurons to damage from oxidative stress. We demonstrate altered mitochondrial number and size, increased mitochondrial ROS production, and altered mitochondrial membrane potential in response to ROS in cells overexpressing RCAN1. Furthermore, RCAN1 overexpression increases cellular susceptibility to oxidative stress by reducing cell viability in response to increasing ROS levels. Our findings have implications for neurodegeneration in DS and AD in which RCAN1, mitochondrial dysfunction, and oxidative stress are all thought to play a pathogenic role.

## 2. Methods

### 2.1. Mice

RCAN1 transgenic (RCAN1^ox^) mice were generated using the human* RCAN1* cDNA encoding the exon 1 splice variant as previously described [6]. Use of mice was approved by the Flinders University and Monash University Animal Welfare Committees. All mice were maintained in the Animal House at the Flinders Medical Centre or Monash University. Mice were fed a normal chow diet and were on a 12-hour light/12-hour dark cycle.

### 2.2. Primary Neuron Culture

For isolation of mouse cortical neurons,pregnant female mice were killed by cervical dislocation at 14 to 16 days after coitus and the foetuses were dissected from the uterine horns in dissecting solution (1.24 mM NaCl, 0.05 mM KCl, 0.01 mM NaH_2_PO4·H_2_O, 0.25 mM HEPES, 0.12 mM MgSO_4_, 0.26% (w/v) D-glucose, 0.3% (w/v) BSA, and pH 7.4). Foetuses were rapidly killed by decapitation and the cerebral cortices were excised and pooled in 15 mL dissecting solution containing 0.25% (w/v) trypsin (Invitrogen, Carlsbad, CA, USA) at 37°C. After 20 minutes, digestion was terminated by the addition of 15 mL dissecting solution containing 0.01% DNase (Sigma-Aldrich, St. Louis, MO, USA). The solution was mixed well and the cells pelleted and resuspended in 2 mL dissecting solution containing 0.02% (w/v) glycine max and 0.004% (w/v) DNase. The cells were dissociated by passing through a blunt-ended glass Pasteur pipette for 2 minutes. Finally, neuronal cells were collected after centrifugation at 500 g for 5 minutes and resuspended in 10 mL plating media (Neurobasal media, Invitrogen, Carlsbad, CA, USA), 10% FCS, 2% B27 supplement, 0.5 mM glutamycin (SAFC Biosciences, Lenexa, KA, USA), and 50 *μ*g/mL gentamicin (Invitrogen, Carlsbad, CA, USA) before counting. Five hours after plating, the media were changed to plating media without FCS. Half the media was changed every two days during culture. All dishes and plates used for culture were treated with 0.005% poly-L-lysine for at least two hours before use (Sigma-Aldrich, St. Louis, MO, USA). To obtain near pure neuronal cultures, 2.5 *μ*g/mL cytosine D-arabinofuranoside (Sigma-Aldrich, St. Louis, MO, USA) was applied to cultures on day four after plating for 24 hours. Experiments were performed on days 8-9 of cultures.

### 2.3. MTT Viability Assay

Primary cortical neurons were grown at a density of 8 × 10^4^ cells/cm^2^ in poly-L-lysine treated plates for 9 days before treatment. PC12 cells were seeded at 4 × 10^5^ cells/well for 6 hours prior to treatment then treated with varying concentrations of H_2_O_2_ for 18 hours. MTT (3-(4,5-Dimethylthiazol-2-yl)-2,5-diphenyltetrazolium bromide) (Sigma) was added at a concentration of 500 *μ*g/mL to each well and incubated at 37°C for 4 hours. Media were discarded and cells were resuspended in 200 *μ*L DMSO by repeated pipetting to solubilize the formazan. Suspensions were transferred to Eppendorf tubes and centrifuged at 12,000 g in a MiniSpin bench top microcentrifuge (Eppendorf) for 4 minutes. 50 *μ*L supernatant was transferred in triplicate to the wells of 96-well plate and absorbance was measured at 590 nm on a Fluostar Optima plate reader (BMG Lab Technologies).

### 2.4. Electron Microscopy

Adrenal medulla was dissected from mice and whole tissue was fixed for 1 hour in EM fixative (4% formaldehyde and 1.5% glutaraldehyde in sodium cacodylate buffer, pH 7.2) and postfixed in 2% osmium tetroxide in sodium cacodylate buffer. Fixed tissue was stained with 2% uranyl acetate and dehydrated through 70%, 90%, and 100% ethanol. Tissue was processed through 1,2-epoxypropane, a 1 : 1 mixture of 1,2-epoxypropane and Procure 812 resin (Electron Microscopy Sciences, USA), and two changes of 100% resin. Tissue and resin were transferred to Beem capsules and placed overnight at 90°C. Survey sections were stained with toluidine blue and 100 nm sections were cut on a Porter-Blum ultramicrotome (Sorvall, USA) using a diamond knife (Micro Star Technologies, USA). Thin sections were stained with Reynold's lead citrate and examined on a Hitachi H-600 transmission electron microscope (Hitachi, Japan). All analysis was done on chromaffin cells photographed at 4000x magnification unless otherwise mentioned. Electron micrographs were analysed by ImageJ image analysis software. Within the adrenal sections, chromaffin cells were distinguished by their characteristic granule morphology and are easily distinguished from cortical cells at the ultrastructural level [38]. Mitochondria were identified by their characteristic, outer and inner membrane, and cristae. Mitochondrial number was calculated utilizing the ImageJ cell counting plugin while mitochondrial area was calculated using the ImageJ free hand application.

### 2.5. Estimation of Mitochondrial Transmembrane Potential

The ratiometric fluorescent dye JC-1 was used to provide relative measurements of mitochondrial membrane potential [39–41]. At depolarized potentials, JC-1 forms monomers producing green fluorescence detectable at 530 nm. At higher membrane potentials, JC-1 forms multimers (J-aggregates) producing red fluorescence that is detectable at 590 nm. Both the monomer and the J-aggregate were excited simultaneously at 488 nm. The ratio of fluorescence emission at 590 : 530 nm was determined as an indicator of the mitochondrial membrane potential [40]. Cultured adrenal chromaffin cells were used for these measurements. Adrenal chromaffin cells were cultured from adult mice as previously described [6, 42]. Briefly, the adrenal medulla was dissected out in cold Locke's buffer (154 mM NaCl, 5.6 mM KCl, 3.6 mM NaHCO_3_, 5.6 mM D-Glucose, 5.0 mM HEPES, and pH 7.4) and incubated with collagenase-A, (Roche, Germany) in Locke's buffer at a concentration of 3 mg/mL, in a shaking water bath at 37°C. The collagenase was diluted in cold Locke's buffer, and cells were pelleted and resuspended in DMEM medium supplemented with 1% penicillin/streptomycin (Invitrogen, Carlsbad, CA, USA) and 10% FCS (JRH Biosciences, Lenexa, USA) and filtered through nylon mesh. Cells were pelleted, resuspended in supplemented DMEM, and plated on 35 mm culture dishes and incubated at 37°C with 5% CO_2_. Cells were maintained in primary culture for 3-4 days prior to experiments. Chromaffin cells were washed twice and incubated in serum free culture media containing JC-1 (0.125 *μ*M) for 30 min. The cultures were then rinsed and left in dye-free media for 20 minutes at 37°C before being subjected to real-time imaging in a Leica SP5 Spectral Confocal Microscope (Leica Microsystems, Wetzlar, Germany). High resolution digital images were analyzed using ImageJ.

### 2.6. Generation of RCAN1-FLAG Fusion Expression Construct

Full length cDNA encoding RCAN1 fused to FLAG was generated by PCR using the following primers: RCAN1-5′-utrF 5′-*gattccgagggggttaacggcgga*-3′ and RCAN1-3′-FLAGR 5′- **** tca***cttgtcatcgtcgtccttgtagtcgctgaggtggatcggcgtgt-*3′ (sequence encoding the FLAG tag is underlined whilst the stop codon is in bold). Template cDNA used in the PCR reaction was generated by RT-PCR of RNA extracted from a Ntera 2/D1 (NT2) human cell line. The resulting 901 bp PCR product had a FLAG tag sequence (DYKDDDDK) incorporated in frame directly upstream of theRCAN1 stop codon and was cloned into pGEM-T Easy (Promega). Sequence analysis carried out using T7 and SP6 primers (Promega) confirmed that the epitope-tag was in frame with the* RCAN1* coding sequence. The 901 bp PCR product was excised from pGEM-T utilizing the* Eco*R1 restriction sites present on either side of the insert. The resulting DNA fragment was ligated into the* Eco*R1 restriction site of pcDNA3 (Invitrogen). As this ligation was not directional, clones were screened by PCR amplification using the T7 forward and the RCAN1-3′-FLAGR primers. Those clones with correct orientation produced a 958 bp PCR product, whilst clones with incorrect orientation produced a 57 bp product. For cell transfection experiments the pcDNA3/RCAN1-FLAG construct was prepared using an endo-free Qiagen Maxi prep. kit. The construct was linearized prior to transfection by digestion with the restriction endonuclease* Pvu*I.

### 2.7. Generation of PC12 Cell Lines Stably Overexpressing RCAN1

7 *μ*g of linearized plasmid DNA was transfected into 1.5 × 10^6^ rat pheochromocytoma PC12 cells by electroporation. As a control, untransfected PC12 cells were grown on a separate plate. To select for transfectants, cells were treated with Geneticin (G418) containing media replaced every 48 hours for 10–15 days or until all the untransfected PC12 cells on the control plate had died. Transfectants were then grown in G418-free media and allowed to form colonies which were picked and subsequently expanded. PC12 cells were cultured in F-12 K containing 10% horse serum, 5% FCS, 1% Pen-Strep (penicillin-streptomycin), and 1% glutamine (Invitrogen, Australia). Cells were grown at 37°C in a 5% CO_2_ humidified environment 95% air and were grown to 80–90% confluency before being passaged. For passaging, cells were treated with 0.125% trypsin (Invitrogen, Australia) for 3 minutes, collected with fresh media, centrifuged at 400 g for 5 minutes, and seeded at a density of 2.5 × 10^5^ cells/mL. Cells were plated out at a density of 2.5 × 10^3^ cells/mL on 35 mm culture dishes and allowed at least 24 hours to adhere to plates before being used for experiments. In selected experiments, 10 mM of N-acetyl-cysteine (Sigma, Australia) was added to culture media for 24 hours before experiments were performed.

### 2.8. RT-PCR and Western Blot Analysis

To test for expression of RCAN1-FLAG, RNA was extracted from cells grown to confluence on a 6 cm plate using TRIzol reagent (Invitrogen). cDNA was generated by RT-PCR using Superscript III enzyme (Invitrogen). PCR reactions were carried out using RCAN1-5′-utrF and RCAN1-3′-FLAGR primers. RCAN1-FLAG protein expression levels were assessed by Western blot analysis. Protein was extracted from PC12 clones and run on a 12% SDS-PAGE gel and transferred onto a PVDF membrane (Immobilon-P, Invitrogen). An *α*-FLAG antibody conjugated to horseradish peroxidase (Sigma) was used to probe the membrane at a dilution of 1 : 1000.

### 2.9. Measurement of ROS Production

Media were removed from PC12 cells in culture dishes and replaced with warm (37°C) Krebs solution (140 mM NaCl, 5 mM KCl, 2 mM CaCl_2_, 1 mM MgCl_2_, 5 mM D-Glucose, 10 mM HEPES, and pH 7.4). MitoSOX mitochondrial superoxide indicator (Invitrogen, Australia) was added to cells at a final concentration of 5 *μ*M. Cells were incubated for 10 minutes at 37°C, protected from light, and then gently washed three times with warm Krebs solution. Fluorescence was viewed using an Olympus IX71 fluorescence microscope (Olympus Ltd., Tokyo, Japan). Images were captured at 20x magnification and at the same exposure time for each experimental group. Images were analysed using Image J analysis software (National Institutes of Health, USA). Background fluorescence of each image was measured in duplicate and removed from the mean fluorescence intensity of cells to obtain final values of mean cell fluorescence intensity.

### 2.10. Statistical Analysis

For individual comparisons, statistical analysis of data was carried out using the *t*-test, two samples assuming equal variances. For multiple comparisons, statistical analysis was performed using an ANOVA followed by a Bonferroni multiple comparison test. All data are expressed as means ± S.E.M. *P* < 0.05 was considered significant.

## 3. Results

### 3.1. Neurons Overexpressing RCAN1 Show Decreased Cell Viability upon Exposure to H_2_O_2_

**Figure 1:**
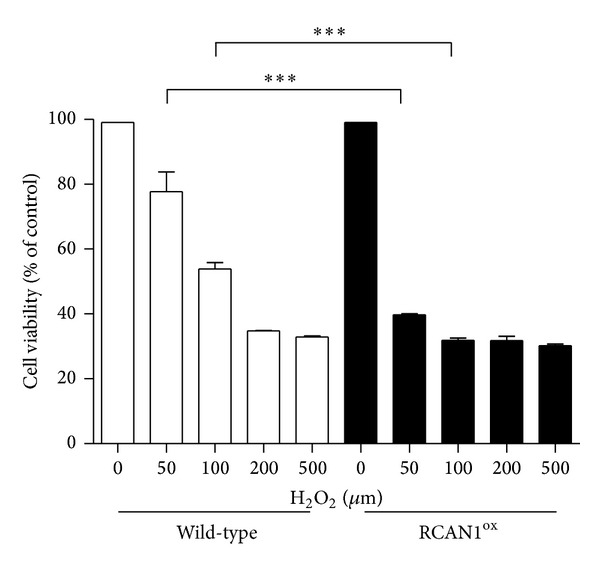
RCAN1^ox^ neurons display decreased viability upon exposure to H_2_O_2_. Primary neuronal cultures from E15 wild-type (light bars) and RCAN1^ox^ mice (dark bars) were exposed to varying concentrations (0–500 *μ*m) of H_2_O_2_ for 18 hours and cell viability was measured via a standard MTT assay. *n* = 4 neuronal cultures from individual wild-type and RCAN1^ox^ mice. Data presented as a percentage of the number of starting viable cells, error bars represent SEM, and ***P < 0.001.

In order to investigate the role of RCAN1 in oxidative stress and cell viability, primary cultures of cortical neurons, prepared from E15 RCAN1^ox^ and wild-type embryos, were treated with varying concentrations of H_2_O_2_ and cell viability was measured by MTT assay after 18 hours. Primary neurons derived from RCAN1^ox^ mice showed a significant (*P* < 0.01) decrease in cell viability at 50 *μ*M H_2_O_2_ with 39.7 ± 0.4% of neurons viable versus 77.7 ± 6.1% of wild-type neurons. At 100 *μ*M H_2_O_2_, there was a larger difference (*P* < 0.0001) in neuronal viability between the two populations; 31 ± 0.6% RCAN1^ox^ neurons were viable versus the 53.9 ± 1.9% of wild-type neurons (Figure 1). Experiments were carried out on three independent cultures with percentage cell viability calculated as a proportion of the viability of untreated cells from the same genotype. Interestingly, there was no difference between the viability of RCAN1^ox^ and wild-type neuronal cultures without H_2_O_2_ treatment.

### 3.2. RCAN1 Regulates Mitochondrial Morphology

**Figure 2:**
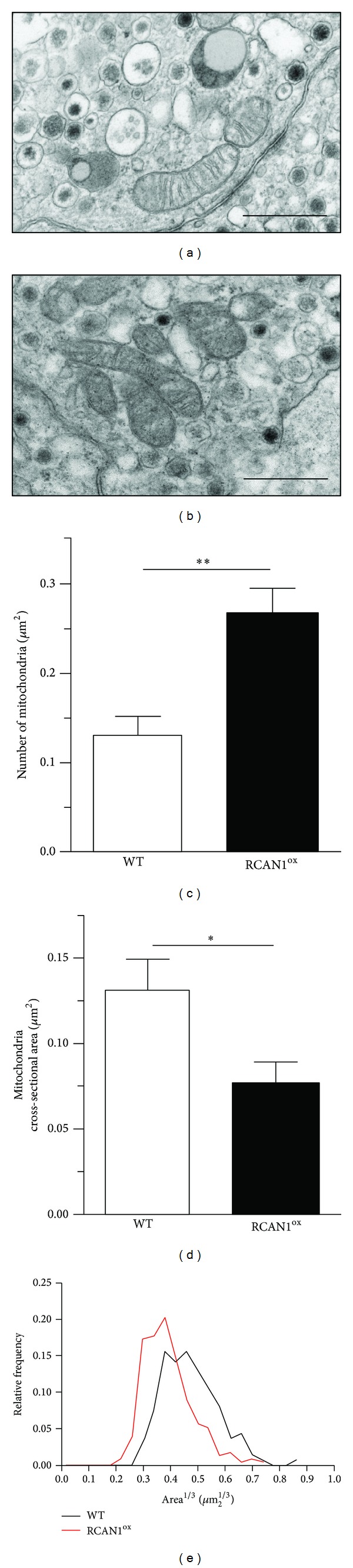
RCAN1 regulates mitochondrial morphology. Representative electron-micrographs from (a) wild-type and (b) RCAN1^ox^ chromaffin cells captured at 4000x magnification (scale bar = 300 nm). RCAN1^ox^ chromaffin cells (black bars) have (c) a greater density of mitochondria and (d) significantly smaller mitochondria when compared to wild-type controls (white bars). (e) RCAN1^ox^ cells (red line) have a higher frequency of smaller mitochondria when compared to wild-type controls (black line). *n* = 9 cells from 3 animals for each genotype, error bars represent the SEM, *P < 0.05, and **P < 0.01.

Alterations in mitochondrial function may underlie the increased susceptibility to oxidative stress observed in primary RCAN1^ox^ neurons. Mitochondrial morphology is regulated by RCAN1 expression in* Drosophila* neurons [37]. We therefore conducted an ultrastructural analysis of WT and RCAN1^ox^ mitochondria in chromaffin cells via electron microscopy. Chromaffin cells are neuroendocrine cells and an accepted model in neuronal studies that we have used previously [6, 7]. Their relative abundance in the adrenal medulla and the ability to obtain these cells in a homogenous population and to readily identify them at an ultrastructural level provide an ideal model for the analysis of mitochondrial size and number via electron microscopy. Mitochondrial appearance was similar, with clearly visible cristae, in both WT and RCAN1^ox^ cells (Figures 2(a) and 2(b)). However, the number of mitochondria was significantly increased (Figure 2(c), *P* < 0.01) and the average mitochondrial area reduced (Figure 2(d), *P* < 0.05) in RCAN1^ox^ cells compared to WT. Analyzing the distribution of mitochondrial area in these cells (Figure 2(e)) demonstrates that RCAN1^ox^ cells had a greater number of smaller mitochondria when compared to wild-type controls. These results are consistent with the observation of Chang and colleagues in* Drosophila*, where the overexpression of RCAN1 resulted in altered mitochondrial morphology due to mitochondrial fission [37].

### 3.3. RCAN1^ox^ Cells Have Altered Mitochondrial Membrane Potential in Response to ROS

**Figure 3:**
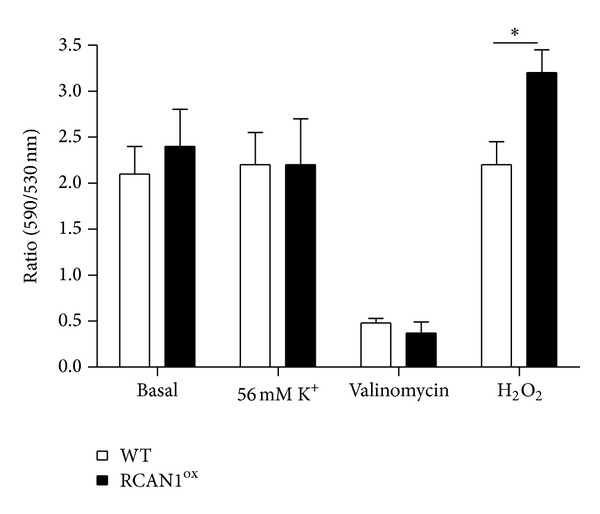
RCAN1^ox^ cells have altered mitochondrial membrane potential in response to ROS. Wild-type (light bars) and RCAN1^ox^ (dark bars) chromaffin cells were stained with the fluorescent mitochondrial membrane potential marker JC-1 under the following conditions: basal (30 cells), 56 mM K^+^ solution (30 cells), 500 nM Valinomycin (10 minutes/10 cells), and 100 *μ*m H_2_O_2_ (60 minutes/9 cells). Error bars represent the SEM; *P < 0.05.

To further ascertain whether mitochondrial function is altered by RCAN1 overexpression, we evaluated mitochondrial membrane potential under various conditions in WT and RCAN1^ox^ chromaffin cells using the fluorescent mitochondrial membrane potential marker, JC-1. We found that mitochondrial membrane potential is similar in both groups under basal conditions, with the mean JC-1 fluorescence ratio being 2.1 ± 0.3 and 2.4 ± 0.4 (Figure 3) in WT and RCAN1^ox^ cells, respectively. No changes in JC-1 fluorescence ratio were observed after depolarization of the plasma membrane using a 56 mM K^+^ solution, demonstrating that the response of JC-1 is not influenced by changes in plasma membrane potential. Depolarization of the mitochondria was achieved by treatment with the K^+^ ionophore Valinomycin (500 nM; 10 minutes) which rapidly reduced the JC-1 ratio to 0.48 ± 0.05 in WT cells and 0.37 ± 0.12 in RCAN1^ox^ cells (Figure 3). However, treatment with H_2_O_2_ (100 *μ*M: 60 minutes) resulted in a significant difference in JC-1 fluorescence between WT and RCAN1^ox^ cells. The change in fluorescence in JC-1 fluorescent ratio between the resting potential in these cells and that obtained after H_2_O_2_ indicated a more severe effect on mitochondrial membrane potential in RCAN1^ox^ cells (2.2 ± 0.3 WT versus 3.2 ± 0.5RCAN1^ox^, *P* < 0.05).

### 3.4. Generation of PC12 Cells Stably Overexpressing RCAN1

**Figure 4:**
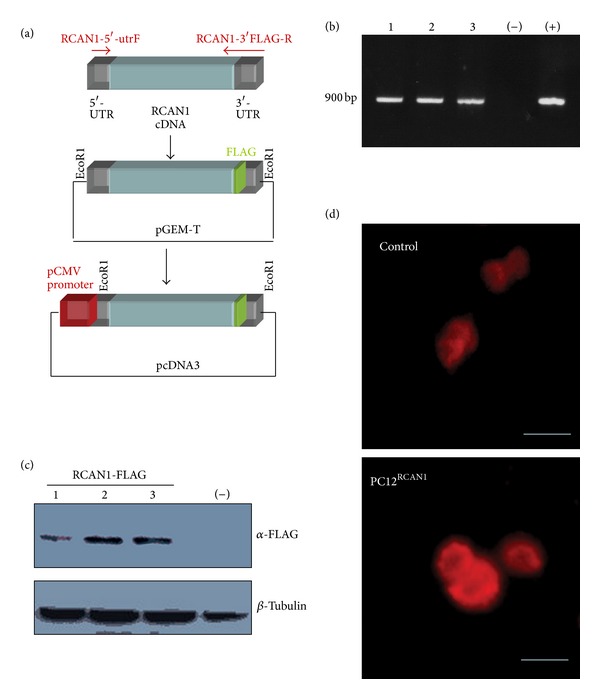
Generation of a stable PC12 cell line overexpressing RCAN1, PC12^RCAN1^. (a) Diagrammatic representation of the generation of the FLAG-fused RCAN1 construct, which was cloned into pGEM-T easy vector and finally ligated to the EcoR1 restriction site of pcDNA3 expression vector containing a pCMV promoter. (b) Agarose gel electrophoresis of the PCR products from the 3 selected clones containing the 900 bp RCAN1-FLAG tag sequence, a water only (−) control, and (+) control using DNA encoding the pGEM-T/RCAN1-FLAG construct. (c) Immunoblot against the FLAG tag in the 3 selected clones and a negative control lacking FLAG normalized to *β*-tubulin loading control. (d) Immunocytochemistry of control PC12 and PC12^RCAN1^ cells with an antibody against RCAN1 demonstrates the elevated RCAN1 expression in PC12^RCAN1^ cells (scale bar = 50 *μ*m).

In order to confirm that the results seen in RCAN1^ox^ cells are not due to changes associated with developmental influences from outside the cells being studied, we developed a PC12 cell line overexpressing RCAN1 (PC12^RCAN1^ cells). Twenty-four independent PC12 clones stably transfected with RCAN1-FLAG (Figure 4(a)) were tested for expression of the construct. All clones generated RNA transcripts of the RCAN1-FLAG construct (three representative clones are shown in Figure 4(b)). Protein expression levels were assessed by Western blot analysis. Of the nine clones tested, three expressed the RCAN1-FLAG at equivalent levels (Figure 4(c)). Untransfected PC12 cells were used as a negative control. Increased RCAN1 protein expression in PC12^RCAN1^ cells was further verified immunohistochemically using an anti-RCAN1 antibody (Figure 4(d)). These results confirmed the stable overexpression of RCAN1 in PC12 cells and were hence used for subsequent experiments.

### 3.5. RCAN1 Overexpression Increases Mitochondrial ROS Production

**Figure 5:**
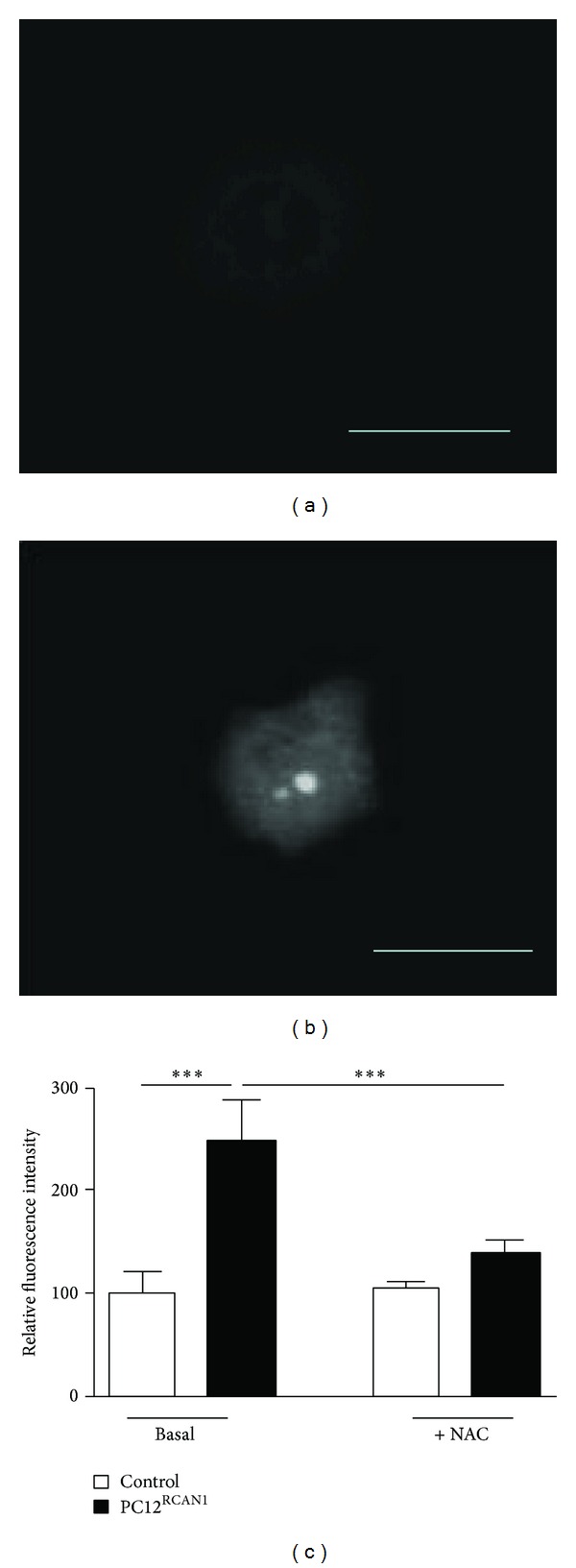
RCAN1 overexpression increases mitochondrial ROS production. Representative images of (a) control and (b) PC12^RCAN1^ cells stained with 100 *μ*M MitoSOX Red at 20x magnification (scale bar = 50 *μ*m). (c) Quantification of MitoSOX fluorescence intensity revealed that control cells (light bars) have significantly less mean fluorescence compared to PC12^RCAN1^ cells (dark bars) under basal conditions. The addition of the antioxidant N-acetylcysteine (NAC) significantly reduces florescence in PC12^RCAN1^ cells while having no effect on control cells. Data has been normalized to basal mean fluorescence in control cells and error bars represent SEM. *n* = 30 cells from each group; ***P < 0.001.

ROS production was measured in control and PC12^RCAN1^ cells using MitoSOX Red, a mitochondrial superoxide indicator (Figures 5(a) and 5(b)). Mitochondrial ROS production was increased in PC12^RCAN1^ cells in comparison to control PC12 cells (Figure 5(c), *P* < 0.001). ROS production was decreased in PC12^RCAN1^cells treated with the antioxidant N-acetylcysteine (NAC) compared to untreated PC12^RCAN1^cells (*P* < 0.001). NAC treatment had no effect on mitochondrial ROS production in control PC12 cells.

### 3.6. PC12^RCAN1^ Cells Are Less Viable upon Exposure to H_2_O_2_

**Figure 6:**
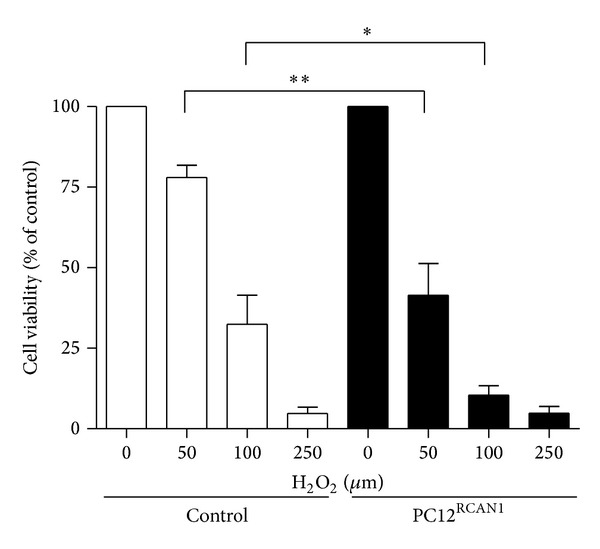
PC12^RCAN1^ cells exhibit reduced viability upon exposure to H_2_O_2_. Control (lights bars) and PC12^RCAN1^ cells (dark bars) were exposed to increasing concentrations of H_2_O_2_ from 0–250 *μ*m for 18 hours. At 50 *μ*M and 100 *μ*M H_2_O_2_, fewer PC12^RCAN1^ cells were viable when compared to control PC12 cells under the same conditions. Data has been normalized to percentage of starting viable cells for each group and error bars represent the SEM. *n* = 13 experiments in control and PC12^RCAN1^ cells; *P < 0.05 and **P < 0.01.

PC12^RCAN1^ cells were subjected to the naturally occurring ROS H_2_O_2_ and viability measured similarly to our experiments in RCAN1^ox^ neurons. PC12^RCAN1^ cells and control PC12 cells were treated with concentrations of H_2_O_2_ ranging from 50 to 250 *μ*M for 18 hours. 41.5 ± 9.8% of PC12^RCAN1^cells were viable after treatment with 50 *μ*M H_2_O_2_ whilst 78.1 ± 3.7% of the control cells remained viable (Figure 6, *P* < 0.01). This difference in cell viability was also observed when cells were treated with 100 *μ*M H_2_O_2_, with 10.5 ± 2.8% of PC12^RCAN1^ cells remaining viable compared to 32.5 ± 9.0% of treated control cells. These results in PC12^RCAN1^ cells confirm that the effect of RCAN1 on cell susceptibility to oxidative stress is due to cell autonomous effects of RCAN1.

## 4. Discussion

This study investigated the role of RCAN1 in regulating mitochondrial function and susceptibility to oxidative stress in a neuronal cell line and primary cells overexpressing RCAN1. Elevated RCAN1 expression in PC12 cells resulted in ROS accumulation, as well as decreased viability in response to H_2_O_2_. Similarly, primary neurons overexpressing RCAN1 had a greater susceptibility to H_2_O_2_-induced oxidative stress. Altered mitochondrial morphology, characteristic of mitochondrial dysfunction, was observed via an ultrastructural analysis of RCAN1^ox^ cells and these cells demonstrate altered mitochondrial membrane potential in response to increased ROS levels. Thus, we demonstrate that overexpressing RCAN1 in primary mammalian neuronal cells causes significant changes to mitochondrial function and oxidative stress responses.

*Nebula*, the* Drosophila melanogaster *ortholog of RCAN1, is localized in mitochondria where it interacts with the ADP/ATP translocator on the inner mitochondrial membrane. The elevated ROS accumulation we observe in PC12^RCAN1^ cells and which has been noted in neurons overexpressing* nebula* [37] may be associated with altered ADP/ATP translocator function resulting in decrease ATP production [37], which in turn could affect oxidative phosphorylation and cause ROS accumulation [43, 44]. The overexpression of RCAN1.1 in ST14A cells also affected the ADP/ATP translocator and mitochondrial permeability transition pore opening, resulting in mitochondrial degradation, autophagy, and reduced cell survival [45]. The altered mitochondrial morphology observed in RCAN1^ox^ chromaffin cells could be related to the regulation of calcineurin by RCAN1 and their various downstream targets. One important calcineurin target is dynamin-related protein 1 (Drp1), a protein which increases mitochondrial fission when dephosphorylated and is dephosphorylated by calcineurin [46]. Increased Drp1 phosphorylation in cells with high RCAN1 expression could potentially underlie the altered mitochondrial morphology observed in RCAN1^ox^ cells.

Elevated RCAN1 expression resulted in decreased cell viability in primary neurons and PC12^RCAN1^ cells in response to H_2_O_2_ exposure. Calcineurin activity is significantly attenuated in response to H_2_O_2_ and the inhibition of calcineurin activity by exogenous pharmacological agents under these conditions increases ROS-induced damage [11]. We postulate that a similar inhibition of calcineurin activity in cells overexpressing RCAN1 results in decreased cell viability. Calcineurin activation plays a central role in controlling either pro- or antiapoptotic cues in different cell types. Calcineurin activity in neurons contributes to apoptosis associated with glutamate excitotoxicity and ionophore-induced Ca^2+  ^ overloading [47, 48] while in cardiac myocytes calcineurin activation imparts a protective effect against H_2_O_2_ induced cell death [49]. These contrasting effects of calcineurin activation highlight the potential complexity of the RCAN1/calcineurin interaction in response to varying stress stimuli. While we have not measured calcineurin activity during this study, our previous work in chromaffin cells indicates that increased RCAN1 expression effects cell functions via inhibition of calcineurin [7]. It is therefore worthwhile to consider that the effects of cell survival observed in cells overexpressing RCAN1 may be a direct effect of the inhibition of calcineurin activity.

Our findings in mammalian cells that RCAN1 overexpression induces multiple facets of mitochondrial dysfunction may have relevance to both DS and AD. In these disorders, neuronal RCAN1 expression is significantly increased [2, 4, 16] and both disorders display a clear profile of neuronal oxidative stress and mitochondrial dysfunction. Neuronal oxidative stress is observed in mouse models of DS [31] and in human DS neurons [29] while mitochondrial function is diminished in this disorder [28]. Similarly, neuronal ROS levels are higher in human tissue from AD patients [32, 33]. Our findings suggest that chronic overexpression of RCAN1 may have negative implications for mitochondrial function and increase mitochondrial ROS production. Furthermore, when cells are presented with an additional insult or challenge such as oxidative stress in AD and DS, the increased RCAN1 levels may further exacerbate this oxidative stress. Indeed, this may be the case in both DS and AD where neurons have chronically elevated levels of oxidative stress [50, 51]. Our finding that the overexpression of RCAN1 regulates mitochondrial morphology and function in mammalian cells therefore provides additional insight into the function of this protein with potential relevance for these related human disorders.

**Figure 7:**
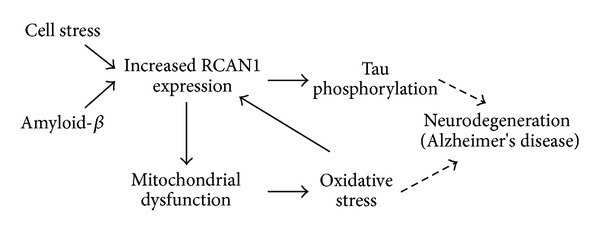
Summative roles of RCAN1 in neurodegeneration and cell function. Elevated amyloid-*β* levels in neuronal cells can lead to elevated RCAN1 expression which inhibits calcineurin activity resulting in accumulation of hyperphosphorylated tau protein. This causes the formation of neurofibrillary tangles that lead to neurodegenerative disorders. We have further shown that elevated RCAN1 expression also leads to mitochondrial dysfunction, resulting in elevated intracellular oxidative stress, both of which are implicated in AD pathogenesis.

Based on our observations in this study and our prior knowledge of RCAN1 function and effectors of RCAN1 expression, we postulate that elevated RCAN1 expression may have a detrimental effect on multiple facets of neuronal function associated with mitochondrial function and AD pathology (Figure 7). Cellular stress, including amyloid-*β* accumulation, can induce RCAN1 expression in neurons, which inhibits the activity of the protein-phosphatase calcineurin. Reduced calcineurin activity leads to the accumulation of hyperphosphorylated tau and subsequent neurofibrillary tangles, a hallmark of AD brains. Further to this, we now show that elevated RCAN1 expression leads to mitochondrial dysfunction including altered mitochondrial morphology and membrane potential and high mitochondrial ROS production. This accumulation of ROS and ensuing oxidative stress can have pathogenic consequences in mammalian neurons that are strongly implicated in AD pathogenesis and also induce a feed forward cycle by increasing RCAN1 expression further. Thus, our data provide a framework for the involvement of RCAN1, mitochondrial dysfunction, and oxidative stress that may be causative in AD progression.
